# The Oldest Homœopathic Physician

**Published:** 1892-01

**Authors:** L. Hoopes

**Affiliations:** West Chester


### THE OLDEST HOMŒOPATHIC PHYSICIAN.

West Chester, October 27th, 1891.

Editor of The Homœopathic Physician:

I notice in The Homœopathic Physician the death of Dr. Lilienthal, and it is stated that he was the oldest living practitioner of Homœopathy in the U. S. If that means he was the oldest man practicing Homœopathy I think it is a mistake, for I think Dr. J. Stuart Leech, of Downington, who is a straight Hahnemannian, is in his eightieth year. He is a graduate of the Jefferson College of Philadelphia and practiced allopathy for nine years after graduation. He is in active practice.

Yours fraternally,

L. Hoopes.

